# Diuretic-Enhanced Dual-Phase F-18 FDG PET/CT: A New Window Into Muscle-Invasive Bladder Cancer

**DOI:** 10.1055/s-0046-1824462

**Published:** 2026-06-17

**Authors:** Cihan Şin, Burcu Esen Akkaş, Özge Vural Topuz, Özgür Omak, Enes M. Kaya, Harun Özdemir, Begüm Çalım Gürbüz, Halil L. Canat, Abdulmuttalip Şimşek, Meryem Kaya

**Affiliations:** 1 Department of Nuclear Medicine, University of Health Sciences, Bağcılar Training and Research Hospital, Istanbul, Türkiye; 2 Department of Nuclear Medicine, University of Health Sciences, Başakşehir Çam and Sakura City Hospital, Istanbul, Türkiye; 3 Department of Nuclear Medicine, Acıbadem Mehmet Ali Aydınlar University, Acıbadem Maslak Hospital, Istanbul, Türkiye; 4 Department of Nuclear Medicine, Hatay Training and Research Hospital, Antakya, Hatay, Türkiye; 5 Department of Urology, University of Health Sciences, Başakşehir Çam and Sakura City Hospital, Istanbul, Türkiye; 6 Department of Pathology, University of Health Sciences, Başakşehir Çam and Sakura City Hospital, Istanbul, Türkiye; 7 Department of Urology, Memorial Bahçelievler Hospital, Istanbul, Türkiye

**Keywords:** bladder cancer, positron-emission tomography, muscle invasion, metabolic tumor volume, total lesion glycolysis

## Abstract

**Objective:**

This study aimed to assess the diagnostic performance of diuretic-enhanced dual-phase F-18 FDG PET/CT in detecting muscle invasion in bladder cancer (BC) and to evaluate the predictive role of metabolic and volumetric PET parameters.

**Methods:**

A total of 105 patients (85 males, 20 females; mean age: 67.8 years) with suspected BC underwent F-18 FDG PET/CT for initial staging. Routine and delayed pelvic imaging was performed after intravenous furosemide. Metabolic and volumetric parameters, including SUV _max_ , SUV _mean_ , metabolic tumor volume (MTV), and total lesion glycolysis (TLG), were measured and correlated with histopathological findings. Statistical analyses consisted of Mann–Whitney U, Kruskal–Wallis, and Receiver operating characteristic (ROC) curve analysis.

**Results:**

Distant metastases were detected in 30 patients (28.5%), which directly influenced treatment decisions. In the TUR-B cohort ( *n* = 75), F-18 FDG PET/CT demonstrated a sensitivity of 78.0%, specificity of 62.5%, positive predictive value of 88.5%, negative predictive value of 43.5%, and an overall accuracy of 74.7% in differentiating malignant from benign bladder lesions. Among the 59 patients with confirmed malignancy, volumetric parameters correlated with pathological T-stage. Patients with T2 tumors had significantly higher SUV _max_ , SUV _mean_ , MTV, and TLG values compared with <T2 tumors ( *p* < 0.001). ROC analysis confirmed the discriminative value of MTV (AUC: 0.814) and TLG (AUC: 0.830), with reliable cutoff values identified.

**Conclusion:**

Diuretic-enhanced dual-phase F-18 FDG PET/CT provides clinically valuable information for the assessment of detrusor muscle invasion and the differentiation of bladder lesions. Quantitative volumetric and metabolic parameters may serve as surrogate biomarkers, supporting preoperative staging and contributing to more precise, personalized treatment strategies for patients with BC.

## Introduction

Bladder cancer (BC) is the most common malignancy of the urinary tract and is four times more frequent in men than in women. 1 Even though the majority of BC is represented by nonmuscle-invasive tumors, 30% of patients have muscle-invasive bladder cancer (MIBC) associated with a high risk of lymph node (LN) involvement and distant metastases that negatively affect survival. 2 Tumor T-staging combined with pathological grade determines the surgical approach for BC patients. The standard treatment approach is transurethral resection (TUR) for non-MIBC and radical cystectomy with pelvic LN dissection for MIBC. 3 BC is diagnosed by histological sampling of the intravesical lesion via cystoscopy. However, evaluating muscle invasion is only possible by sampling the muscularis propria via TUR. 4

Given the significant impact of MIBC in treatment and the potential risks associated with invasive TUR procedures, the need for an accurate and noninvasive grading method before treatment is crucial for personalized management.

Although BC is a high FDG-avid tumor, the physiological excretion of FDG via kidneys and the accumulation of FDG in the bladder complicates the detection of primary tumor in the bladder wall. For this reason, NCCN guidelines only recommend FDG PET/CT for screening distant metastases in the initial staging of patients with invasive BC. 5

Delayed FDG PET/CT pelvic images following intravenous diuretic administration are a reliable approach for detecting bladder tumors while both reducing the concentration of F-18 FDG in urine and increasing the FDG uptake in the tumor. 6 7 Metabolic parameters such as SUV _max_ (maximum standardized uptake value), total lesion glycolysis (TLG), and metabolic tumor volume (MTV) obtained from staging F-18 FDG PET/CT imaging have been shown to have correlation with both LN and distant metastases in many cancers including urinary tract carcinomas. 8 9 In endometrial cancer patients, TLG and MTV have been shown to correlate with intense myometrial invasion, which has a negative prognostic impact and affects patient management. 10 However, research regarding the predictive value of these parameters for muscle invasion in BC remains limited.

In this prospective study, we aimed to investigate the contribution of diuretic-enhanced delayed F-18 FDG PET/CT imaging in the detection of muscle invasion status in tumoral lesions in patients diagnosed with primary BC. We hypothesized that accurate preoperative detection of muscle-invasive tumors by noninvasive diagnostic methods would allow radical cystectomy to be considered as the primary surgical procedure, instead of TUR followed by subsequent radical surgery.

## Materials and Methods

This study was designed prospectively in accordance with the principles of the Declaration of Helsinki, the Patients' Rights Regulation, and ethical guidelines. The study was initiated following approval from the Ethics Committee of İstanbul Başakşehir Çam and Sakura City Hospital, dated January 31, 2024, and numbered E-96317027–514.10–235703716, and informed consent was obtained from all participants.

## Patient Selection and Preparation

### Inclusion Criteria

Cystoscopic examination and/or imaging modalities were used to establish a preliminary diagnosis of BC in a total of 105 patients (85 males [81.8%] and 20 females [18.2%]), who were subsequently included in the study. All patients underwent F-18 FDG PET/CT imaging at the Department of Nuclear Medicine for initial diagnosis and staging.

Patients younger than 18 years, those with a prior history of malignancy, those who had undergone urinary tract surgery, as well as patients with diabetes mellitus or active inflammatory diseases that could affect FDG uptake, were excluded from the study.

### F-18 FDG PET/CT Imaging Protocol

After fasting for at least 6 hours, patients whose blood glucose levels were confirmed to be below 200 mg/dL received an intravenous injection of F-18 FDG (3.7 MBq/kg). Routine imaging was performed 60 minutes postinjection. To further reduce physiological urinary activity, 20 mg of furosemide was administered intravenously as a slow infusion to patients without contraindications such as impaired renal function, electrolyte imbalance, or cardiac insufficiency. Patients were encouraged to drink 1 L of water orally to ensure hydration. Blood pressure was monitored at 15-minute intervals, during which patients were asked to urinate.

Sixty minutes after diuretic administration, delayed pelvic imaging covering the bladder was performed while patients had a full bladder (partially cleared of radioactivity).

### F-18 FDG PET/CT Imaging Details

Imaging was performed using a Philips Ingenuity TF PET/CT Time-of-Flight system PET and CT scanners. Patients were positioned supine, and scans were acquired from the vertex to the mid-thigh.

Low-dose CT imaging was performed with 120 mA and 130 keV settings for attenuation correction and anatomical correlation before PET imaging. Images were evaluated visually and semiquantitatively in three planes (coronal, transverse, sagittal).

Using the CT images, three-dimensional manual volumes of interest (VOIs) were drawn for each lesion in axial, sagittal, and coronal planes to include the entire mass. Healthy bladder tissue and FDG activity in urine were excluded from these VOIs.

From the routine whole-body and delayed pelvic PET/CT images, the extent of disease was assessed. Delayed pelvic imaging was specifically used for the metabolic evaluation of the primary bladder lesion. 11 A PET/CT-positive bladder lesion was defined as a focal area of increased FDG uptake visually higher than the surrounding background activity, corresponding to a bladder wall lesion on CT images, and demonstrating an SUV threshold of ≥ 2.5 12 For quantitative analysis, regions of interest were manually drawn around the lesion on delayed pelvic images using Philips IntelliSpace Portal. During segmentation, only focal uptake corresponding to the bladder wall lesion was included on fused PET/CT images, while urinary activity within the bladder lumen was manually excluded. MTV was defined as the volume of tumor tissue demonstrating FDG uptake above an SUV threshold of 2.5. Maximum standardized uptake value (SUV _max_ ) and mean standardized uptake value (SUV _mean_ ) were recorded for each lesion. TLG was calculated as the product of MTV and SUV _mean_ , according to the formula: TLG = MTV × SUV _mean_ .

After routine preoperative workup including PET/CT, all patients were scheduled for surgery following international procedure guidelines.

### Pathological Evaluation

Postoperative pathological examination was performed by a team of pathologists specialized in bladder tumors. They evaluated tumor type, grade, stage, lamina propria invasion, muscularis propria invasion, perivesical fat invasion, prostatic stromal invasion, presence of carcinoma in situ, lymphovascular invasion, perineural invasion, presence of necrosis, and surgical margin status in detail.

Tumor TNM staging was assessed based on the 2017 AJCC TNM classification. Patients with postoperative pathology indicating malignancy were classified as the malignant lesion (ML) group, while those with benign results were classified as the benign lesion (BL) group. Malignant cases were further categorized as T2 (muscle-invasive) or <T2 (nonmuscle-invasive).

Based on the findings of F-18 FDG PET/CT imaging, patients were stratified into two groups according to the presence of distant metastases: Group 1 comprised patients with distant metastatic involvement, whereas Group 2 included those without evidence of distant metastasis.

### Statistical Analysis

Statistical analysis was performed using IBM SPSS Statistics 22.0. Descriptive statistics included frequency, percentage, mean, and standard deviation. Data were presented as mean ± standard deviation.

When parametric assumptions were not met, the Mann–Whitney U test was used to compare means between two independent groups, and the Kruskal–Wallis test was used for comparisons among more than two independent groups. Receiver operating characteristic (ROC) curve analysis was performed in the entire TUR-B cohort ( *n* = 75) to evaluate the diagnostic performance of PET-derived parameters for predicting muscle-invasive disease. Optimal cut-off values were determined using the Youden index. Sensitivity, specificity, and their 95% confidence intervals were calculated. A *p* -value < 0.05 was considered statistically significant.

## Results

A total of 105 patients (85 males [81.8%], 20 females [18.2%]) who were scheduled for surgery with a preliminary diagnosis of BC were included in the study. Ages ranged from 32 to 92 years, with a mean age of 67.8 years ( Table 1 ).

**Table 1:** Patient demographics and clinical characteristics

Variable	*n* (%)/mean ± SD
Sex	
Male	85 (81%)
Female	20 (19%)
Age (y)	
Male	67.6 ± 12.49
Female	68.8 ± 11.57
Distant metastasis on PET-CT	
Absent	75 (71.5%)
Present	30 (28.5%)
Type of operation	
TUR-B	75 (63%)
Histopathology	
Malignant	59 (78.6%)
Benign	16 (21.4%)
Histopathological subtype	
Urothelial carcinoma	57 (96.6%)
Adenocarcinoma	1 (1.7%)
Small cell neuroendocrine carcinoma	1 (1.7%)
TUR-B T stage	
Ta	31 (52.5%)
T1	9 (15.2%)
T2	19 (32.3%)

Note: Values are presented as mean ± SD or *n* (%). *p* < 0.05 was considered statistically significant.

In preoperative F-18 FDG PET/CT scans, distant organ metastases were detected in 30 of the 105 patients (28.5%), including the lungs (13 patients), bones (12 patients), and liver (five patients). These patients were considered inoperable, and their treatment plans were modified accordingly: chemotherapy ( *n* : 30) and radiotherapy ( *n* : 10) were administered.

Among patients in Group 1, the primary tumor exhibited significantly elevated metabolic parameters, including SUV _max_ , SUV _mean_ , MTV, and TLG, compared with those in Group 2 ( *p* = 0.04).

Seventy-five patients underwent TUR-B operation within 3 weeks (range: 1–6) following the F-18 FDG PET/CT scan. Postoperative histopathology revealed malignant disease in 59 patients ( *n* : 78.6%), whereas the results were compatible with benign disease in 16 patients (21.4%). Volumetric PET parameters such as SUV _max_ (11.4 vs. 3.4, *p* = 0.001), SUV _mean_ (6.1 vs. 1.9, *p* = 0.001), MTV (12.7 vs. 8.5, *p* = 0.007), and TLG (115.9 vs. 51, *p* = 0.004) were significantly higher in patients with histopathologically proven BC compared with benign disease ( Table 2 ). Among the 75 patients who underwent TUR-B operation, F-18 FDG PET/CT demonstrated a sensitivity of 78.0%, specificity of 62.5%, positive predictive value of 88.5%, negative predictive value of 43.5%, and an overall accuracy of 74.7% in distinguishing malignant from benign bladder lesions.

**Table 2:** Comparison of PET/CT metabolic and volumetric parameters between patients with benign and malignant bladder lesions

	SUV _max_ (g/mL)	SUV _mean_ (g/mL)	MTV (cm ^2^ )	TLG
Benign ( *n* = 16)	3.47 ± 5.99 (0–21)	1.98 ± 3.46 (0–12)	8.56 ± 21.27 (0–80)	51.08 ± 128.32 (0–456)
Malignant ( *n* = 59)	11.46 ± 8.80 (0–30)	6.19 ± 4.72 (0–20)	12.74 ± 17.43 (0–100)	115.98 ± 193.84 (0–1,000)
*p* -Value	0.001	0.001	0.007	0.004

Note: Data are shown as mean ± SD unless otherwise indicated. *p* < 0.05 indicates a statistically significant difference.

Pathological evaluation of 16 patients with BL post-TUR-B revealed chronic inflammation ( *n* = 11), granulomatous inflammation ( *n* = 2), urothelial hyperplasia ( *n* = 2), and urothelial papilloma ( *n* = 1). Granulomatous lesions showed significantly higher SUV _max_ , SUV _mean_ , MTV, and TLG values compared with other BL ( *p* = 0.008).

**Fig. 1:**
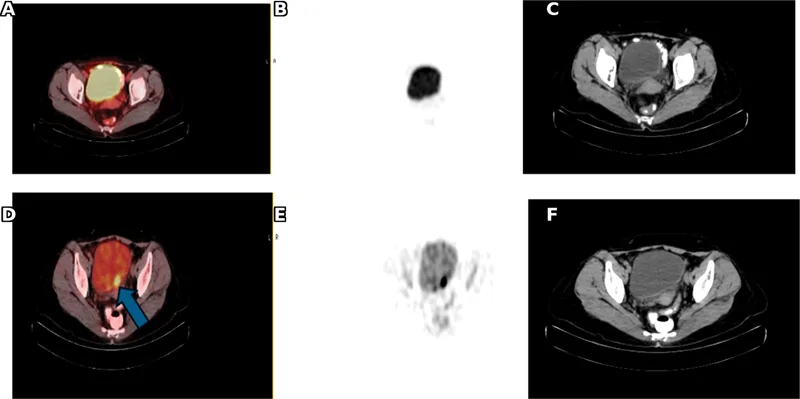
Representative case of nonmuscle-invasive bladder cancer (BC; Ta) on diuretic-enhanced dual-phase F-18 FDG PET/CT. ( **A–C** ) Routine PET/CT images acquired 60 minutes after FDG injection demonstrate bladder wall thickening in the left posterior wall; however, tumor-related FDG uptake is not clearly distinguishable due to intense physiological urinary activity. ( **D–F** ) Delayed PET/CT images obtained after intravenous diuretic administration demonstrate focal FDG uptake in the lesion following reduction of urinary activity. Quantitative PET parameters were relatively low (SUV _max_ : 7, SUV _mean_ : 4, MTV: 5 cm ^3^ , TLG: 20). Histopathological evaluation confirmed Ta-stage nonmuscle-invasive BC.

In the cohort of 59 patients with histopathologically confirmed malignant pathology following TUR-B, metabolic and volumetric PET parameters demonstrated statistically significant variation across different pathological T stages ( *p* < 0.001). The highest values for parameters such as SUV _max_ , SUV _mean_ , MTV, and TLG were observed in patients with muscle-invasive (T2) tumors, whereas the lowest values were recorded in those with Ta-stage lesions ( Fig. 1 ).

Among the patients with malignant pathology ( *n* = 59), 19 were classified (32.3%) as having muscle-invasive tumors (T2), whereas the remaining 40 patients (67.7%) belonged to the nonmuscle-invasive group (<T2).

**Fig. 2:**
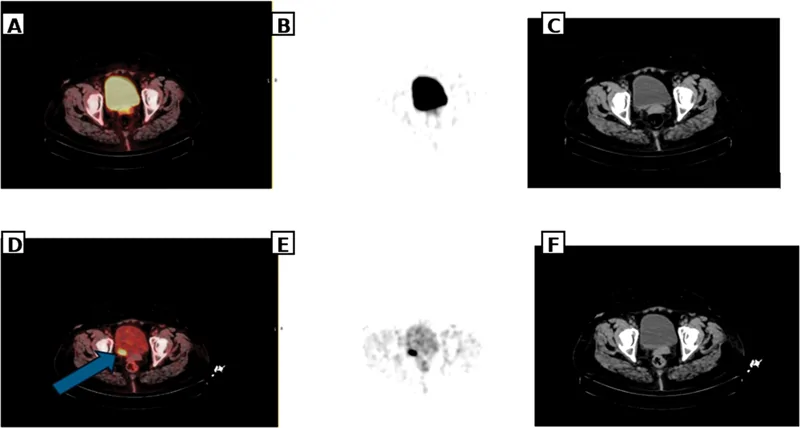
Representative case of muscle-invasive bladder cancer (BC; T2) on diuretic-enhanced dual-phase F-18 FDG PET/CT. ( **A–C** ) On routine PET/CT images obtained 60 minutes after FDG injection, bladder wall thickening is visible in the right posterolateral wall; however, tumor-related FDG uptake is not clearly distinguishable because of intense urinary activity. ( **D–F** ) On delayed PET/CT images acquired after diuretic administration, focal pathological FDG uptake corresponding to the bladder wall lesion is clearly visualized following reduction of urinary activity (SUV _max_ : 13, SUV _mean_ : 7, MTV: 10 cm ^3^ , TLG: 70). Histopathological evaluation confirmed T2-stage muscle-invasive BC.

In the T2 group, the volumetric PET/CT parameters such as SUV _max_ (16.2 vs. 7.5, *p* < 0.001), SUV _mean_ (8.8 vs. 4.1, *p* < 0.001), MTV (25.1 vs. 7.3, *p* < 0.001), and TLG (249.1 vs. 52.2, *p* < 0.001)were significantly higher than in the <pT2 group ( Table 3 ; Fig. 2 ).

**Table 3:** Volumetric PET/CT parameters in patients with T2 and <T2 bladder tumors

Parameter	T2 ( *n* = 19)	<T2 ( *n* = 40)	*p* -Value
SUV _max_	16.2 ± 7.4 (0–30)	7.5 ± 8.2 (0–30)	<0.001
SUV _mean_	8.8 ± 4.2 (0–20)	4.1 ± 4.3 (0–17)	<0.001
MTV (cm ^2^ )	25.1 ± 24.9 (0–100)	7.3 ± 12.7 (0–80)	<0.001
TLG	249.1 ± 287.4 (0–1,000)	52.2 ± 89.6 (0–456)	<0.001

Note: Data are shown as mean ± SD. *p* < 0.05 indicates a statistically significant difference.

**Fig. 3:**
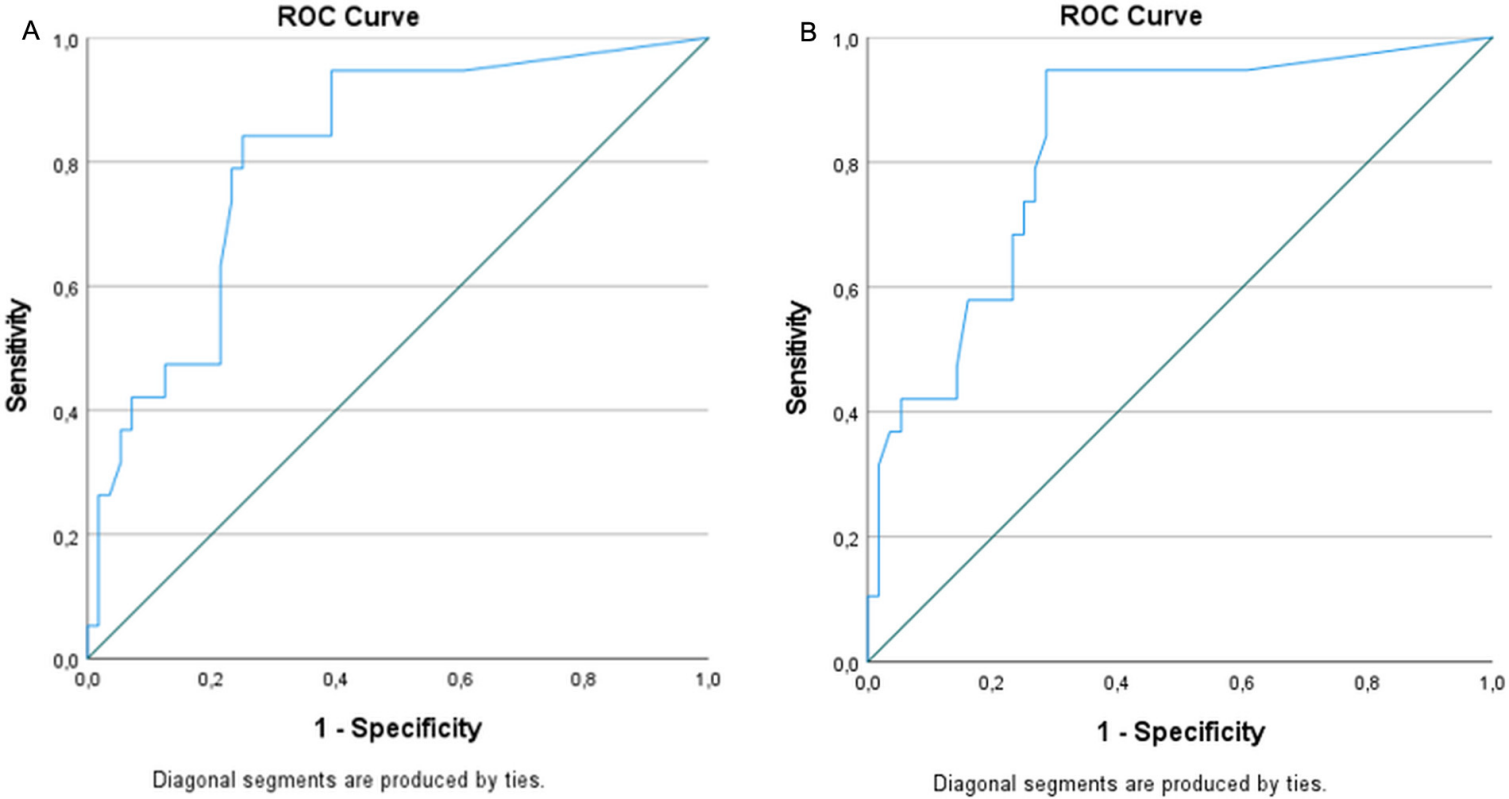
( **A** ) ROC curve analysis of MTV for predicting T2 tumors in patients undergoing TUR-B showed an AUC of 0.814 (95% CI: 0.69–0.94). An MTV cutoff value > 8.15 predicted T2 tumors with 85% sensitivity and 75% specificity. ( **B** ) ROC curve analysis of TLG for predicting T2 tumors in patients undergoing TUR-B showed an AUC of 0.830 (95% CI: 0.71–0.95). A TLG cutoff value > 52.30 predicted T2 tumors with 95% sensitivity and 72% specificity.

ROC curve analysis of MTV for predicting T2 tumors in patients undergoing TUR-B showed an AUC of 0.814 (95% CI: 0.69–0.94). An MTV cutoff value > 8.15 predicted T2 tumors with 85% sensitivity and 75% specificity ( Fig. 3A ).

ROC curve analysis of TLG for predicting T2 tumors in patients undergoing TUR-B showed an AUC of 0.830 (95% CI: 0.71–0.95). A TLG cutoff value > 52.30 predicted T2 tumors with 95% sensitivity and 72% specificity ( Fig. 3B ).

## Discussion

Accurate assessment of muscle invasion represents a fundamental step in the management of BC, as it significantly influences therapeutic decision-making and patient prognosis. Patients with NMIBC can often be treated effectively with TUR of the bladder tumor (TUR-B) followed by intravesical therapy. In contrast, muscle invasion constitutes an adverse prognostic factor that is associated with increased recurrence rates and reduced survival. For MIBC, the standard of care remains radical cystectomy combined with pelvic lymphadenectomy, yielding an overall survival rate of approximately 50%. 3 13

Given the prognostic implications of muscle invasion, its early and reliable identification is crucial for implementing personalized treatment strategies. The use of the standardized Vesical Imaging-Reporting and Data System has high sensitivity and specificity in detecting MIBC. 12 However, even with the improved magnetic resonance imaging (MRI) technology, which provides almost excellent soft tissue resolution, neglecting the need for TUR before radical surgery based on MRI findings is a debated issue. 14

The correlation of various quantitative tumor parameters such as MTV and TLG with aggressive tumor features and advanced tumor stages has been demonstrated in a variety of malignancies. Likewise, several studies have suggested that FDG PET/CT can be beneficial for selecting high-risk BC patients preoperatively. However, its clinical role in risk stratification of such patients is not well defined. Furthermore, the value of MTV and TLG and the corresponding optimal cutoffs for the prediction of tumor stage and prognosis are largely unexplored.

In this study, we observed a statistically significant positive correlation between volumetric metabolic parameters and tumor size, which corresponded to higher pathological T stages. Elevated SUV _max_ , MTV, and TLG values were indicative of increased tumor burden and biological aggressiveness, supporting their role as potential surrogate biomarkers for muscle invasion in BC. In the TUR-B cohort, these parameters were lowest in Ta-stage tumors and highest in T2-stage tumors, highlighting their clinical relevance in accurate preoperative T-stage stratification.

In the present prospective study, we considered that these volumetric quantitative tumor parameters obtained from the staging PET/CT may have a role in diagnosing patients with MIBC and help guide personalized treatment in patients with BC. Due to the scarcity of existing data, we sought to establish preoperative cutoff values using diuretic-enhanced F-18 FDG PET/CT. We observed that both MTV and TLG were significantly elevated in the muscle-invasive group. Specifically, an MTV > 8.15 cm ^3^ and TLG > 52.3 were associated with high diagnostic accuracy for detecting T2 disease.

In clinical practice, F-18 FDG PET/CT is primarily utilized for staging locally advanced disease following TUR-B, identifying distant metastases, and detecting locoregional or systemic recurrence during follow-up. 15 Notably, F-18 FDG PET/CT has demonstrated superiority over conventional radiologic methods in the detection of distant metastases. 16 In accordance with previous studies, our analysis revealed distant organ metastases in 28.5% of patients during initial staging, leading to significant alterations in therapeutic planning. 17

In fact, BC is an FDG-avid tumor and a recent meta-analysis reported the pooled sensitivity and specificity of FDG PET/CT in detecting BC as 80 and 84%, respectively. Despite these figures, F-18 FDG PET/CT does not outperform cross-sectional imaging modalities such as CT or MRI in the evaluation of primary bladder tumors and therefore remains inadequate for accurate local (T) staging. 18 Accordingly, its utility is largely restricted to the assessment of metastatic disease in the setting of MIBC. At present, MRI and computed tomography (CT) remain the most widely employed conventional imaging modalities for local (T) staging of BC. Takeuchi et al demonstrated that diffusion-weighted MRI provides sufficient tissue contrast to differentiate muscle-invasive (T2) lesions from nonmuscle-invasive tumors (Tis, Ta, T1). 19 Conversely, while CT imaging has proven effective in identifying advanced-stage disease (T3/T4), it lacks the spatial and soft tissue resolution necessary to reliably discriminate between T1 and T2 lesions. 20

The role of F-18 FDG PET/CT in local T-staging remains limited, partially due to the excretion of FDG into the urinary tract, which can obscure visualization of bladder wall lesions. To overcome this issue, forced diuresis and delayed imaging have been shown to improve the diagnostic performance of FDG PET/CT. In a prospective study conducted by Nayak et al, diuretic-enhanced F-18 FDG PET/CT demonstrated superior diagnostic accuracy compared with contrast-enhanced CT, particularly in the detection of primary bladder tumors and regional LN metastases. These findings suggest that incorporating a forced diuresis protocol may enhance the performance of F-18 FDG PET/CT in the local staging of BC and offer a more reliable alternative to conventional imaging modalities. 11 In our study, consistent with previously reported literature, furosemide-induced diuresis combined with delayed pelvic F-18 FDG PET/CT imaging effectively reduced urinary tracer accumulation within the bladder, thereby enhancing the delineation of bladder tumors from adjacent tissues ( Fig. 2 ).

In this study, we evaluated the diagnostic performance of F-18 FDG PET/CT in differentiating malignant from benign bladder lesions. Our findings demonstrated a sensitivity of 78%, specificity of 62.5%, positive predictive value of 88.5%, negative predictive value of 43.5%, and an overall accuracy of 74.7%. These results suggest that PET/CT is a valuable diagnostic tool, particularly for confirming malignancy, although its limited specificity and negative predictive value indicate restricted reliability in excluding BL. 21 In this prospective study, histopathological examination revealed benign pathology in 16 patients. Among this group, two patients had granulomatous diseases that mimicked malignancy in both MR and PET/CT images with significantly higher metabolic and volumetric parameters compared with the others. Ma et al reported that granulomatous reactions developing following intravesical Bacille Calmette-Guérin therapy for BC show similarities to malignant neoplastic processes on radiological imaging, complicating differential diagnosis and posing significant challenges in clinical management. 22

Our study demonstrated a statistically significant positive correlation between volumetric and metabolic parameters derived from PET imaging and tumor size, which corresponded to higher pathological T stages. Elevated SUV _max_ , MTV, and TLG values were indicative of increased tumor burden and biological aggressiveness, supporting their role as potential surrogate biomarkers for muscle invasion in BC. In the TUR-B cohort, these parameters were lowest in Ta-stage tumors and highest in T2-stage tumors, highlighting their clinical relevance in accurate preoperative T-stage stratification. A meta-analysis by Lu et al confirmed the diagnostic efficacy of F-18 FDG PET/CT for metastatic disease, yet emphasized the paucity of evidence supporting its role in detecting detrusor muscle invasion in contrast to the results of our study. 23 Also, in a prospective evaluation, Sharma et al analyzed dynamic F-18 FDG PET/CT imaging at various time points but found no consistent correlation between SUV _max_ and tumor stage (Ta–T4). 24 Similarly, Li et al reported limited sensitivity of F-18 FDG PET/CT in detecting Ta–T3 tumors, with relatively improved performance in T4 lesions among 73 patients undergoing preoperative evaluation prior to radical cystectomy. 25 We considered that the use of diuretic-enhanced delayed pelvic images as the standardized study protocol contributed to the higher detection rates of T2 tumors in the study group. The prospective study design enabled systematic data acquisition and longitudinal follow-up, thereby improving the reliability and clinical relevance of prognostic PET/CT parameters. To our knowledge, this investigation represents one of the largest prospective studies focused specifically on F-18 FDG PET/CT–derived volumetric and metabolic parameters in the preoperative assessment of muscle-invasive BC. Accurate discrimination between nonmuscle-invasive and muscle-invasive BC is clinically critical, as the treatment strategies for these two groups differ substantially. 3 In current practice, local staging is based primarily on histopathological evaluation of TURBT specimens and multiparametric MRI 20 ; however, understaging and delays in definitive treatment remain important clinical challenges. In this context, our study showed that MTV, TLG, and SUV-based parameters derived from delayed diuretic-enhanced FDG PET/CT may contribute to identifying patients with a high likelihood of detrusor muscle invasion. Nevertheless, these parameters should not be considered replacements for TURBT or MRI, but rather adjunctive decision-support tools that may help guide the need for re-TURBT, earlier multidisciplinary evaluation, and definitive treatment planning, particularly in cases with equivocal local staging.

Although the present study is a prospective one, there are some limitations. The relatively small sample size, single-center design, and uneven distribution of tumor stages and histological subtypes may have negatively contributed to the results. Moreover, variability in VOI delineation—particularly in irregular lesions or those adjacent to normal tissue—may have impacted the accuracy of volumetric measurements. A limitation of our study is that VOIs were manually drawn in three planes by a single reader, and inter-observer reproducibility was not assessed. Therefore, potential variability related to observer dependency could not be fully excluded.

## Conclusion

Our study demonstrates that diuretic-enhanced dual-phase F-18 FDG PET/CT may provide complementary information in the staging of patients with known or suspected BC, not only for the detection of distant metastases but also for identifying patients at higher risk of muscle-invasive disease. PET-derived metabolic and volumetric parameters may contribute to preoperative risk stratification and treatment planning. However, these findings require validation in larger prospective studies with long-term follow-up before their role in routine clinical practice can be more clearly established.
